# Treatment of Intercondylar Humeral Fractures With 3D-Printed Osteosynthesis Plates

**DOI:** 10.1097/MD.0000000000002461

**Published:** 2016-01-22

**Authors:** Feng Shuang, Wei Hu, Yinchu Shao, Hao Li, Hongxing Zou

**Affiliations:** From the Department of Orthopedics, the 94th Hospital of Chinese People's Liberation Army, Changcheng Hospital Affiliated to Nanchang University, Nanchang, China.

## Abstract

The aim of the study was to evaluate the efficacy custom 3D-printed osteosynthesis plates in the treatment of intercondylar humeral fractures.

Thirteen patients with distal intercondylar humeral fractures were randomized to undergo surgery using either conventional plates (n = 7) or 3D-printed plates (n = 6) at our institution from March to October 2014. Both groups were compared in terms of operative time and elbow function at 6 month follow-up.

All patients were followed-up for a mean of 10.6 months (range: 6–13 months). The 3D-printing group had a significantly shorter mean operative time (70.6 ± 12.1 min) than the conventional plates group (92.3 ± 17.4 min). At the last follow-up period, there was no significant difference between groups in the rate of patients with good or excellent elbow function, although the 3D-printing group saw a slightly higher rate of good or excellent evaluations (83.1%) compared to the conventional group (71.4%).

Custom 3D printed osteosynthesis plates are safe and effective for the treatment of intercondylar humeral fractures and significantly reduce operative time.

## INTRODUCTION

Intercondylar humeral fractures are severe injuries of the elbow and are difficult to treat. In China, the incidence of intercondylar humeral fractures is increasing, with often greater severity, possibly due to the rapidly growing transportation and construction industries. Most patients, in our experience, are injured via high-energy trauma that frequently leads to comminuted fractures and joint surface damage. The most widely used treatment of intercondylar humeral fractures includes open reduction, internal fixation, and rehabilitation.^1^ However, due to the anatomy of the distal humerus, the incidence of postoperative complications is high and includes elbow dysfunction, nonunion, and deformity.^2^

In recent years, the use of 3D printing has allowed for the rapid manufacturing of custom-designed implants for orthopedics and reconstructive surgery, often achieving better treatment outcomes than current mainstays.^3–7^ This study was designed to investigate the feasibility, efficacy, and safety of 3D printed osteosynthesis plates in the treatment of intercondylar humeral fractures.

## MATERIALS AND METHODS

### Patients

From March to October 2014, 13 patients with distal intercondylar humeral fractures (AO type C3) were treated at our hospital. The mechanisms of injury were falls from height in 5 patients and traffic accidents in 8 patients. The patients all underwent surgery and were randomly divided into 2 groups, a conventional plate group (n = 7) and a 3D-printed plate group (n = 6). All surgeries were performed by the same team, all aspects of this study were approved by the Ethics Committee of the 94th Hospital of the Chinese People's Liberation Army, and informed consent was obtained from all patients before inclusion.

### Surgical Procedure

**FIGURE 1:**
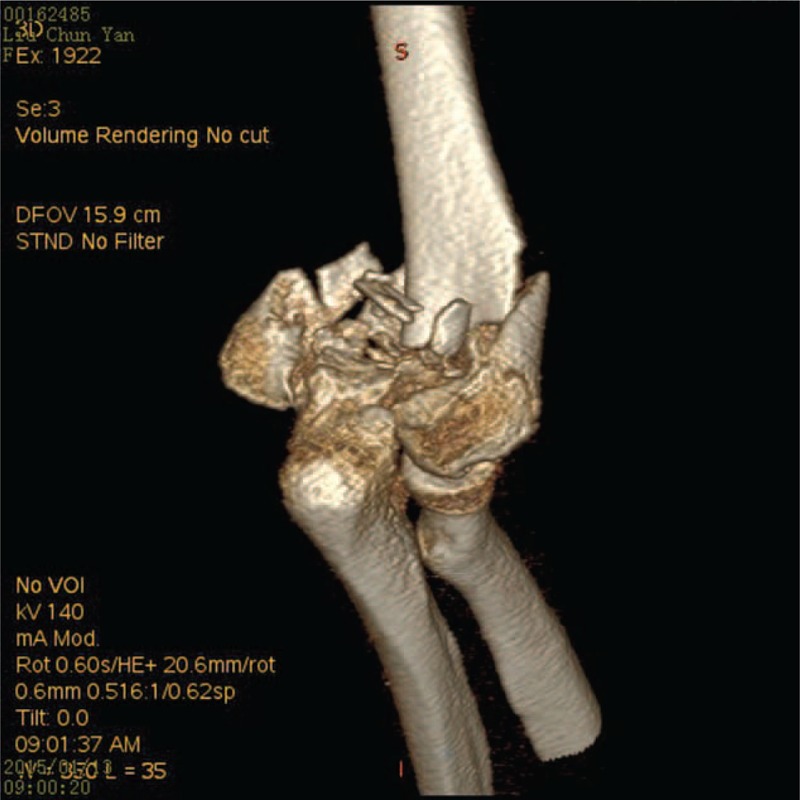
3D reconstructed computed tomography images of a 53-year old woman with a comminuted intercondylar humeral fracture of the right arm.

All fractures were evaluated using 3D reconstructed computed tomography (CT) (Figure 1). Each patient was placed into the supine position and the brachial plexus was blocked. A combined approach through the lateral and medial sides of the triceps brachii muscle was used. The triceps were dissected between the lateral and medial humeral condyles, and the triceps tendon was exposed. The triceps tendon was pulled medially and the lateral column was reduced under direct visualization and fixed with plates. The triceps tendon was then pulled laterally and the intercondylar fracture was reduced and fixed with cannulated screws. The medial column was also reduced and fixed with plates.

### 3D Printing

**FIGURE 2:**
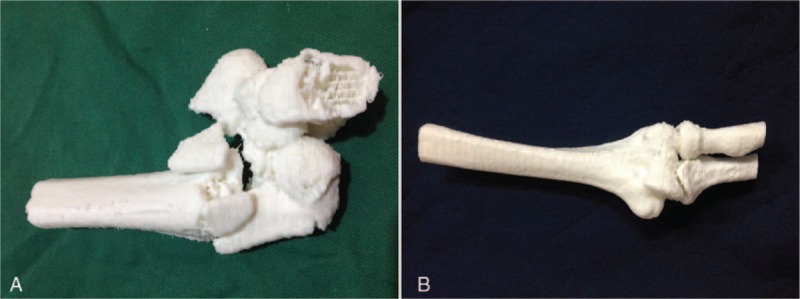
3D printed models of the injured elbow (A) and the mirrored contralateral elbow (B).

The humeri of both the injured and contralateral sides were scanned with CT (1 mm slice intervals) and saved using the DICOM 3.0 format. The data were then transferred to Mimics v.11.1 software (Materialise, Ann Arbor, MI) for design of the plate. The *Region Growing* command was used to establish the Mask of the humerus. The pixel set of the humerus was processed using the *Calculate 3D form mask* command to produce the mirror image of the contralateral side, which was used as the 3D model of the injured side. The Mask pixel set of each fragment was established and the 3D Object was calculated using the Mask. The 3D model of the injured elbow was produced using Unite Boolean calculation. The design data was then transferred to the 3D printer (SRP400B, Huasen 3D Printing Research, Changzhou, China) and exact 1:1 models of the injured elbow and the mirrored contralateral elbow were fabricated (Figure 2). The surface of the 3D model was then polished using the Smooth function of the FEA module to reduce the signal noise in the 3D images.

### Simulation of Reduction

**FIGURE 3:**
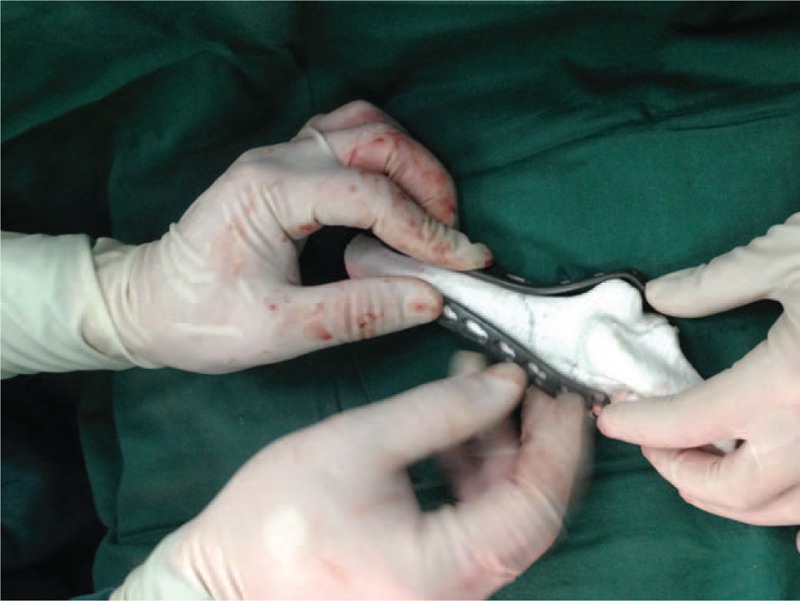
The optimal site for plate implantation was determined using the 3D printed model.

The 3D model of each patient's fractured elbow was slowly rotated to simulate the intraoperative reduction maneuver. The lateral and medial columns of the distal humerus were re-established. The carrying angle was restored to that of the contralateral side. Osteosynthesis plates with the proper size and number of holes were then fabricated using the 3D printer. The optimal implantation site of the printed plate was then determined using the models (Figure 3).

### Postoperative Evaluation

Bone union was evaluated by an independent observer using X-rays and 3D reconstructed CT. Plaster casts were used for 2 weeks in 2 patients in the conventional group and in 1 patient in the 3D-printing group with severe comminuted fractures. The remaining 10 patients were encouraged to being early exercises of a continuous passive motion of the upper limb. An independent observer evaluated the range of motion using a goniometer at each month's follow-up visit. Range of motion of the injured elbow was assessed in each patient using the Mayo elbow performance score (MEPS) system and classified as excellent (≥90 points), good (75–89 points), fair (60–74 points), and poor (<60 points).^8,9^

### Statistical Analysis

Continuous data are presented as mean ± standard deviation. Categorical data are presented as frequencies. Comparisons were made using Student's *t* test or a chi-square test. All statistical analyses were performed using SPSS v17.0 software (SPSS, Chicago, IL). *P* < 0.05 was considered statistically significant.

## RESULTS

There were 6 patients in the 3D-printing group, including 4 men and 2 women, with a mean age of 46.2 ± 11.6 years (range: 31–62 years). The conventional plate group had 7 patients, including 6 men and 1 woman, with a mean age of 40.3 ± 10.9 years (range: 27–59 years). No significant differences in sex, age, and injury type were observed between the 2 groups (*P* > 0.05). All 13 patients were followed-up for a mean of 10.6 months (range: 6–13 months).

**FIGURE 4:**
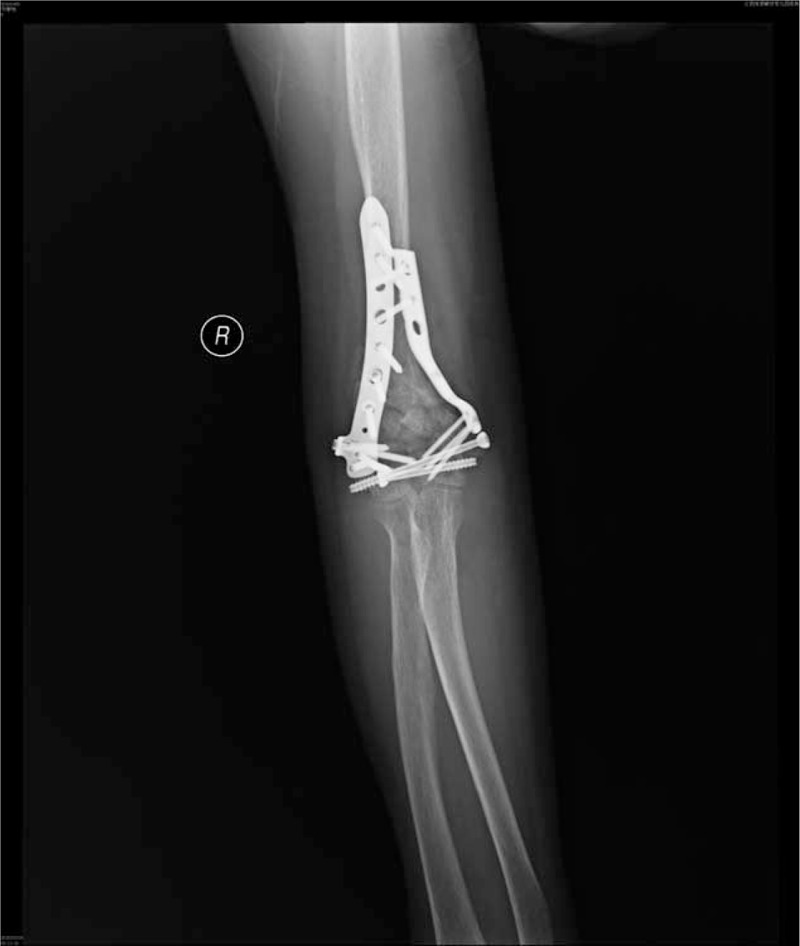
X-ray image showing callus formation at 6 weeks postoperative.

Operative time was significantly shorter in the 3D-printing group (70.6 ± 12.1 min) than in the conventional group (92.3 ± 17.4 min, *P* = 0.026). The mean time to bone union was 3.4 months. At the last follow-up period, bone union was seen in all patients (Figure 4). In the 3D-printing group, elbow function was scored as excellent in 3 patients, good in 2 patients, and fair in 1 patient. In the conventional group, elbow function was scored as excellent in 2 patients, good in 3 patients, fair in 1 patient, and poor in 1 patient. The 2 groups showed no significant difference in the rate of patients with excellent or good elbow function, although the 3D-printing group saw a slightly higher rate of good or excellent evaluations (83.1%) compared to the conventional group (71.4%). Unsurprisingly, the ranges of elbow flexion/extension and pronation/supination did not differ significantly between the 2 groups (*P* *>* 0.05, Table 1).

**TABLE 1:**
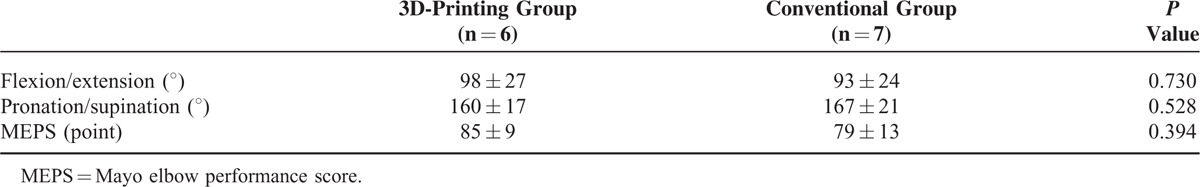
Range of Motion of the Elbow (Mean ± Standard Deviation)

One patient in the conventional group experienced intraoperative traction injury of the ulnar nerve. The injury resolved after 3 months of rehabilitation. No wound infections or other complications were observed.

## DISCUSSION

Distal humeral fractures account for 2% of total adult fractures, of which ∼1/3 are intercondylar fractures.^10^ Although intercondylar fractures are rare, they present a clinical challenge due to the complex anatomy and the associated high incidence of severe trauma. Humeral intercondylar fractures are often caused by the impact of the trochlear notch of the ulna, leading to the separation and dislocation of the bilateral condyles.^11^ The articular surface is often damaged in humeral intercondylar fractures and therefore accurate reduction and stable fixation are essential for satisfactory functional recovery of the elbow, especially for type C3 comminuted fractures.^12,13^ If accurate reduction and stable fixation are achieved, the patient should be able to initiate early exercise. In our study, all patients received stable fixation. Three patients with severe comminuted fractures were managed with plaster casts for 2 weeks and all showed good functional recovery after removal of the casts.

In the conventional group, it was difficult to completely evaluate the fracture fragments and the reduction was time-consuming and technically difficult. The osteosynthesis plates often did not fit the fracture well and needed to be bent or cut to achieve an adequate fit. In addition, accurate screw insertion was difficult. These problems, however, were significantly mitigated with the use of the 3D-printed plates. Computer-aided navigation can also help improve surgical accuracy, but its application is restricted by the complexity of the operation and the associated high equipment costs. On the contrary, 3D printing technology is convenient and practical. 3D printing has some of the advantages of navigation while providing custom fabricated plates for each patient.

3D printing is extremely helpful for preoperative evaluation and planning as well as for intraoperative navigation. Using 3D printing, the 1:1 size model of the injured elbow helped in evaluating the fracture type and fragments and improved the accuracy of reduction.^14^ Additionally, the mirrored model of the contralateral elbow was useful for surgical planning and could also be used for practice and teaching.^15,16^ The custom made osteosynthesis plates was also fit to each patient's specifications in order to facilitate uncomplicated surgical placement. This avoids the additional shaping and bending that was required with the conventional plates. In addition, the software used allowed for the simulation of implant positioning and screw placement. This level of simulation may account for the reduced operative duration seen in the 3D printed group and may additionally help reduce intraoperative bleeding leading to better functional recovery. Our study showed that the mean operative time was significantly shorter in the 3D-printing group than in the conventional plate group. However, the 2 groups did not differ significantly in elbow function at the last follow-up period. We speculate that this might be attributable to the relatively small sample size and the short follow-up period. In both groups, the flexion/extension range of motion of the elbow was <100° and may be the result of insufficient rehabilitation.

Despite these promising results, there are still some limitations of 3D-printed osteosynthesis plates. Mainly, they require time to prepare and print, and therefore are not compatible with emergent cases. Additionally, in patients with severely comminuted fractures, the current technology is unable to differentiate the smallest fracture fragments. Furthermore, the 3D printing technology used in our study is based on CT images which lack the information on the adjacent soft tissue and vasculature. Also, the printed 3D models of the fracture sites are still quite different from what is seen in the patient during surgery and may compromise the accuracy of plate implantation.

## CONCLUSION

We propose a novel method for the preparation of 3D printed models of intercondylar humeral fractures and osteosynthesis plates. This method is both safe and effective for the treatment of adults with intercondylar humeral fractures and has a significantly shorter operative time compared to conventional plates.
